# ATP binding by an F_1_F_o_ ATP synthase ε subunit is pH dependent, suggesting a diversity of ε subunit functional regulation in bacteria

**DOI:** 10.3389/fmolb.2023.1059673

**Published:** 2023-02-27

**Authors:** Alexander Krah, Timothy Vogelaar, Sam I. de Jong, Jolyon K. Claridge, Peter J. Bond, Duncan G. G. McMillan

**Affiliations:** ^1^ Korea Institute for Advanced Study, School of Computational Sciences, Seoul, South Korea; ^2^ Bioinformatics Institute, Agency for Science, Technology and Research (A*STAR), Singapore, Singapore; ^3^ Department of Biotechnology, Delft University of Technology, Delft, Netherlands; ^4^ School of Fundamental Sciences, Massey University, Palmerston North, New Zealand; ^5^ Department of Biological Sciences, National University of Singapore, Singapore, Singapore

**Keywords:** F1Fo ATP synthase, regulation-physiological, alkaliphile bacteria, aerobe, polyextreme environments

## Abstract

It is a conjecture that the ε subunit regulates ATP hydrolytic function of the F_1_F_o_ ATP synthase in bacteria. This has been proposed by the ε subunit taking an extended conformation, with a terminal helix probing into the central architecture of the hexameric catalytic domain, preventing ATP hydrolysis. The ε subunit takes a contracted conformation when bound to ATP, thus would not interfere with catalysis. A recent crystallographic study has disputed this; the *Caldalkalibacillus thermarum* TA2.A1 F_1_F_o_ ATP synthase cannot natively hydrolyse ATP, yet studies have demonstrated that the loss of the ε subunit terminal helix results in an ATP synthase capable of ATP hydrolysis, supporting ε subunit function. Analysis of sequence and crystallographic data of the *C. thermarum* F_1_F_o_ ATP synthase revealed two unique histidine residues. Molecular dynamics simulations suggested that the protonation state of these residues may influence ATP binding site stability. Yet these residues lie outside the ATP/Mg^2+^ binding site of the ε subunit. We then probed the effect of pH on the ATP binding affinity of the ε subunit from the *C. thermarum* F_1_F_o_ ATP synthase at various physiologically relevant pH values. We show that binding affinity changes 5.9 fold between pH 7.0, where binding is weakest, to pH 8.5 where it is strongest. Since the *C. thermarum* cytoplasm is pH 8.0 when it grows optimally, this correlates to the ε subunit being down due to ATP/Mg^2+^ affinity, and not being involved in blocking ATP hydrolysis. Here, we have experimentally correlated that the pH of the bacterial cytoplasm is of critical importance for ε subunit ATP affinity regulated by second-shell residues thus the function of the ε subunit changes with growth conditions.

## Introduction

F-type ATP synthases synthesize ATP, the universal energy source in most living cells. The enzyme consists of a membrane embedded F_o_ domain, which is composed of the membrane embedded proteolipid ring (*c* subunits), the collar-like *a* subunit which is asymmetrically wrapped around the *c*-ring, and the *b* subunit dimer which links the membrane embedded *c*-subunit ring and *a* subunit to the F_1_ domain. The catalytic component of the F_1_ domain (Stock et al., 1999) consists of the asymmetric hexameric α_3_β_3_ assembly. The asymmetry is caused by the central stalk γ subunit, which is bound to the ε subunit (in bacteria), or the δ subunit (in mitochondria) (Abrahams et al., 1994). The δ subunit (bacteria) (Sobti et al., 2016) or oligomycin sensitivity conferral protein (OSCP) (mitochondria) (Rees et al., 2009) connects the *b* subunit dimer with the hexameric α_3_β_3_ assembly. Recent structural studies describing the F_o_ and F_1_ domains have been released (Hahn et al., 2016; Hahn et al., 2018; Guo et al., 2019; Guo et al., 2021; Demmer et al., 2022), providing reliable structural information about the whole enzyme complex. ATP synthases are driven by an electrochemical (H^+^ or Na^+^) (Mitchell, 1961; Dimroth, 1997) gradient, which enforces a rotation in the membrane embedded domain (Sambongi et al., 1999). The rotation of the *c*-ring induces a conformational change (Böckmann and Grubmüller, 2002; Kubo et al., 2022) in the soluble F_1_ domain, triggering the catalytic synthesis of ATP (Hutton and Boyer, 1979). Under certain cellular conditions, most F_1_F_o_ ATP synthases can perform the inverse reaction, i.e., ATP hydrolysis for pH homeostasis or to extrude excess Na^+^ (Mitchell, 1961; Dimroth, 1997).

**FIGURE 1:**
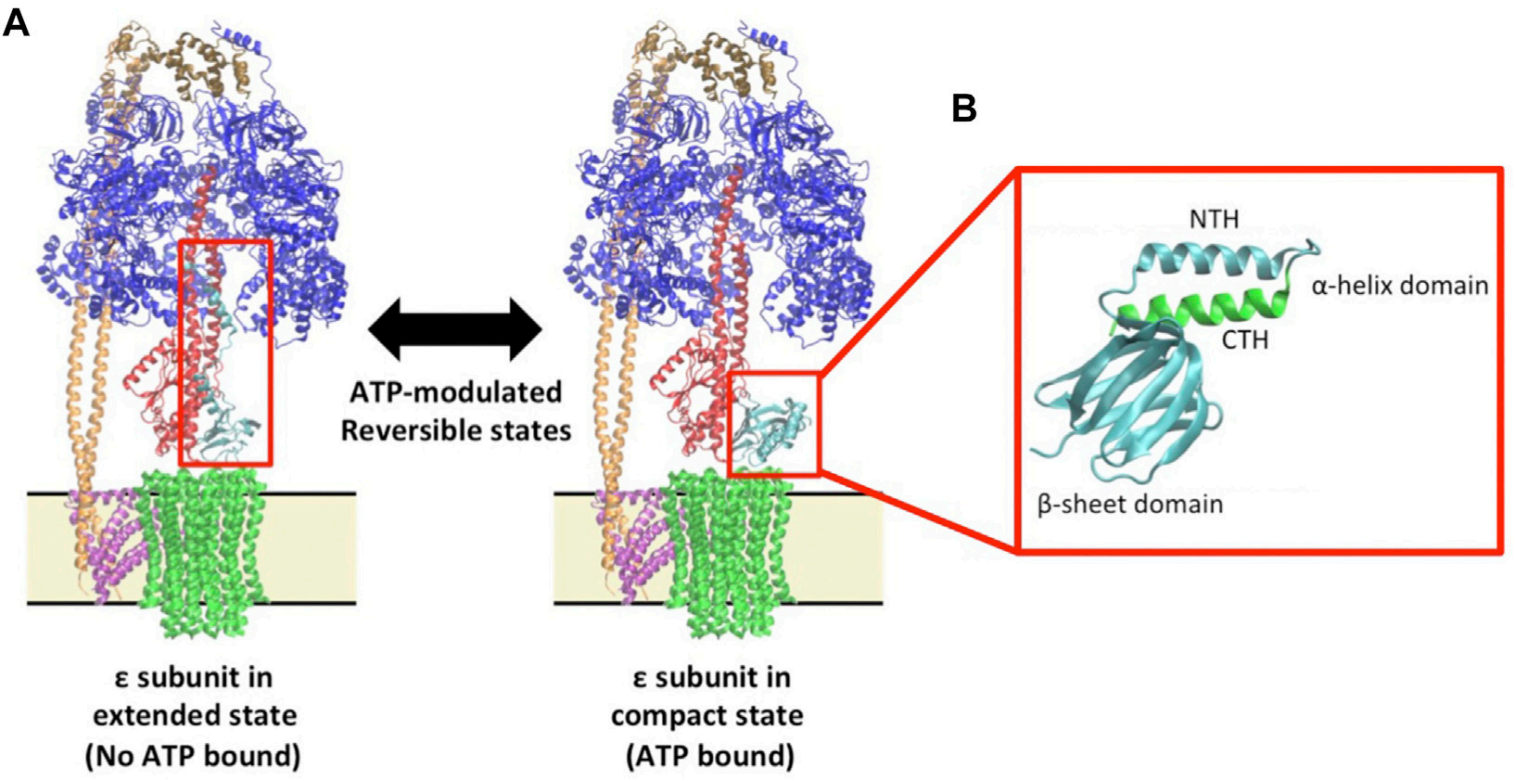
Epsilon structure and function in the context of the F_1_F_o_ ATP synthase **(A)** The *Escherichia coli* F_1_F_o_ multi-subunit complex protein is displayed showing a structure with the ε; subunit in the extended ‘up-state’ in which ATP is not bound (Left: 5T4O) and a structure with the with ε; subunit in the compact ‘down-state’ in which ATP is bound (Right: 5T4O, exchanging chain H with 1AQT). The F_1_ subunits, α_3_β_3_γδε, are shown in blue, blue, red, brown and aqua, respectively, and the membrane-bound F_o_ subunits, *ab*_2_ *c*_10_, are shown in pink, orange, and green respectively. Membrane boundaries around the F_o_ are indicated by a yellow box bound by black lines. The red boxes encompass the ε; subunit in either state. **(B)** A zoom in of the ε; subunit in the “down-state” showing the sheet and helical domains. For clarity of relative orientation, the N-terminal helix (NTH) and β-sheet domain are shown in cyan while the C-terminal helix (CTH) is shown in green.

ADP/Mg^2+^ inhibition is a common ATPase inhibition mechanism in mammals (Drobinskaya et al., 1985) and bacteria (Hyndman et al., 1994; Hirono-Hara et al., 2001; McMillan et al., 2016). This inhibition partially prevents the hydrolysis of ATP at homeostatic pH to a species-dependent extent (Cingolani and Duncan, 2011; Zarco-Zavala et al., 2014). The notable exception to this is the *Caldalkalibacillus thermarum* F_1_F_o_ ATP synthase that has been demonstrated to be a *physiologically* non-reversible enzyme (Cook et al., 2003; McMillan et al., 2007). It is important to note that ADP/Mg^2+^ inhibition has long been proposed to be relieved by the addition of lauryldimethylamine-oxide (LDAO) detergent (Cook et al., 2003; McMillan et al., 2007; McMillan et al., 2016), which serves to “enhance or unlock” ATP hydrolysis activity, even in the *physiologically* non-reversible *C. thermarum* enzyme (McMillan et al., 2007). In addition to ADP/Mg^2+^ inhibition, organisms from different domains of life have developed unique mechanisms to control this wasteful hydrolysis of ATP. In mammals, the pH dependent (Cabezón et al., 2000) inhibitory protein IF_1_ (Pullman and Monroy, 1963) regulates ATP hydrolysis activity (Cabezon et al., 2000). In contrast, in a number of model bacteria, the ε subunit has been a long-standing candidate for regulation of ATP hydrolytic function (Krah, 2015; Krah et al., 2018). The mechanism behind this regulation has been proposed to proceed *via* a conformational change in the ε subunit structure (Suzuki et al., 2003). The structure of the ε subunit includes two c-terminal helices that can either be parallel to each other in a compact conformation (Yagi et al., 2007) (also referred to as the “down-state”), or can adopt an extended conformation (also referred to as the “up-state”; see Figure 1) (Krah et al., 2018). Conversely, the up-state has been proposed to be inhibitory to ATP hydrolytic function (Cingolani and Duncan, 2011; Shirakihara et al., 2015), and is tightly coupled to proton transport ability when the cellular ATP concentration falls below a certain threshold (Feniouk et al., 2007; Kadoya et al., 2011). The ε subunit is able to switch from an “up-state” to a “down-state” through the binding of ATP/Mg^2+^ (i.e.,: there is now much more ATP in the cell). This is thought to allow the enzyme to then perform ATP hydrolysis. Binding of ATP over other nucleotides is very specific (Kato et al., 2007; Kato-Yamada, 2016) The structural basis for selectivity was recently simulated, indicating a perturbed binding network between protein and ligand when ATP is replaced with GTP (Krah et al., 2020). Last, in mycobacteria it has been shown that an extension of the α subunit inhibits ATP hydrolysis (Wong and Grüber, 2020; Wong et al., 2022).

However, despite this seemingly clear division in regulation between higher animal life and bacteria, ε subunit-mediated regulation apparently does not occur in α-proteobacteria, which use a regulatory mechanism governed by the unique ζ subunit (Zarco-Zavala et al., 2014; Krah et al., 2018). The ζ subunit has been proposed to share a similar mechanism to IF_1_, and for this reason, the model organism *Paracoccus denitrificans* has been proposed to be an evolutionary bridge between higher-order life and bacteria (Morales Rioz et al., 2010; Zarco-Zavala et al., 2020).

To date, structural and functional studies suggest that ε subunit ATP binding have been dependent on the conditions under which the experiment was conducted, and from which bacterium the F_1_F_o_ ATP synthase originated (Krah et al., 2018). The ε subunit from various organisms has been resolved in the down-state when being bound to ATP (Yagi et al., 2007; Ferguson et al., 2016; Sobti et al., 2019) or in the absence of ATP (Wilkens et al., 1995; Yagi et al., 2010; Joon et al., 2018; Guo et al., 2021; Shin et al., 2022). The ATP binding affinities of isolated ε subunits from different organisms range from the micro-to the milli-molar range; *Bacillus* PS3 (*K*_*d*_ = 4.3 μM) (Kato et al., 2007), *Bacillus subtilis* (*K*_*d*_ = 2.1 mM) (Kato-Yamada, 2005; Krah et al., 2021) or *Escherichia coli* (*K*_*d*_ = 22 mM) (Yagi et al., 2007). This wide range over three orders of magnitude suggests different physiological functions and regulation of the ε subunit in different organisms. Interestingly, the ε subunit of *M. tuberculosis* did not bind to ATP at the conditions measured (Biukovic et al., 2013).

At face value, given that the bulk-phase ATP concentration in *E. coli* cells is on average 1.5 mM (Yaginuma et al., 2014), and 9.6 mM in the glucose fed state (Bennett et al., 2009) in accordance with their ATP *K*_*d*_ values (Yagi et al., 2007; Kato et al., 2007), the ε subunit from *E. coli* and *Bacillus* PS3 should theoretically adopt an up- or down-state, respectively, in bulk-phase cell homeostasis. This is in complete agreement with a recent finding by Milgrom and Duncan (2020) who demonstrated that over 50% of the *E. coli* enzymes are in the ε subunit extended conformation (Milgrom and Duncan, 2020). Yet despite this, extensive growth studies by Taniguchi *et al* using an *E. coli* ε subunit C-terminal helix (CTH) mutant (ΔCTH; effectively removing half of the ATP binding site, see Figure 1B) demonstrated that under a wide range of nutrient limited conditions, the removal of the CTH had no measurable effect on growth rate, molar yield, membrane potential, or intracellular ATP concentration. The pH of the growth media was also decreased to 5.0, where the cell would be theoretically stressed enough to require the ATP synthase to pump out protons to maintain cell pH homeostasis, but this also had no detectable effect (Taniguchi et al., 2011). This suggests that at least under the conditions examined, the CTH and ATP binding of the ε subunit in *E. coli* is dispensable for growth and survival. This study also suggests that additional compensatory mechanisms may be at work in the cellular environment–the obvious mechanistic suggestion being ADP/Mg^2+^ regulation. Single molecule measurements may be useful to reveal such mechanisms if conducted under physiologically relevant conditions (Duncan et al., 2014).

However, this may not be the situation in *Bacillus* sp., where to date the *K*_*d*_ for ATP of the ε subunit is in bulk-phase cytoplasmic range (Kato-Yamada, 2005; Kato et al., 2007; Krah et al., 2021). Keis *et al* demonstrated in the *C. thermarum* ε subunit that either mutation of a group of positively changed amino acids to alanine (εR116A, εH117A, εK118A, εR119A, εR123A, and εR127A; referred to as “ε6A”) or complete removal of the CTH (ΔCTH) resulted in a fully reversible enzyme from an enzyme previously incapable of ATP hydrolysis. Interestingly, in a mutant enzyme with 4 of the ε6A mutations (εR116A, εH117A, εK118A, εR119A; ‘ε4A’) the *C. thermarum* enzyme was not reversible, implying the arginine residues of the ε subunit at positions 123 and 127 have a critical role in non-reversibility (Keis et al., 2006). It should be noted that this study was conducted in both the F_1_ domain alone, and a complete reconstituted F_1_F_o_ enzyme. These data are *clearly* in support of a regulatory role for the ε subunit. Conversely, a recent crystallographic study using the F_1_ domain found that in the presence or absence of ATP, the *C. thermarum* structure was in the down-state (Ferguson et al., 2016), suggesting that the *ε* subunit has no regulatory role. In this study, an N-terminal helix (NTH) mutant (εD89A, εR92A; see Figure 1B) resulted in a lack of ability to bind ATP and was shown to be in the “down-state” (Ferguson et al., 2016). As with the *E. coli* studies of Milgrom and Taniguchi previously mentioned, these results are seemingly in conjecture with each other. In this case it is even more pertinent to note that a role for the ε subunit as a regulator in non-reversible ATP synthase would be seemingly of utmost importance, where the energetic cost of unregulated ATP hydrolysis would be highest due to the highly alkaline oligotrophic environment where *C. thermarum* is found (pH 9.5/65 °C) (McMillan et al., 2009; de Jong et al., 2020). However, both this study and an extensive single molecule study (McMillan et al., 2016) suggest ADP/Mg^2+^ to be the main mode of ATP hydrolytic inhibition in this enzyme. Clearly, there is more to the conundrum of ε subunit function that has not yet been revealed, and the role of the physiological environment needs to be further considered, along with the environmental pressures that the organism faces.

When considering physiological environment, while ATP binding has been extensively studied, the roles of physiologically relevant cell cytoplasmic pH on ε subunit ATP binding has been largely overlooked *in vitro*. This seems to be at odds with the intention of examining physiological function considering that the proposed core role of ATP hydrolysis by the F_1_F_o_ ATP synthase is pH homeostasis. Furthermore, the binding site residues of ε subunits from both *Bacillus* PS3 and *B. subtilis* are seemingly identical when examining primary sequence, despite having orders of magnitude different ATP binding affinity (Kato-Yamada, 2005; Kato et al., 2007; Krah et al., 2021). This binding affinity difference has been proposed to be induced by an allosteric Mg^2+^ binding site (Krah and Takada, 2015). However, recently a residue outside the *B. subtilis* ε subunit ATP binding site, εE102, was shown to have profound influence on ATP binding affinity. This was proposed to be through sequestering εR99 away from the binding site building a salt bridge between εR99 and εE102. Indeed, when the charge was removed by mutation to alanine (εE102A) a ∼10 fold increase in ATP binding affinity was observed. However, more strikingly, a change to a positive charge *via* mutation to arginine (εE102R) resulted in a ∼54 fold increase in ATP binding affinity, presumably through repulsion causing rotation of εR99 towards the active site (Krah et al., 2021). In addition, ATP binding studies have revealed that the εR103A/εR115A double-mutant of *Bacillus PS3* binds ATP with a two orders of magnitude increase in affinity (52 nM) compared to wild-type (4.3 μM) (Kato-Yamada, 2016). We proposed that this increase in affinity is caused by an enhanced hydrogen bonding network and a loss of repulsive contacts between the Mg^2+^ ion and basic protein residues with other positively charged residues at the binding site (Krah and Takada, 2016; Krah et al., 2017). Taken together, this suggests that “second shell” residues outside the ATP binding site are capable of influencing what occurs in the nucleotide binding site through either Mg^2+^, direct amino-acid hydrogen bonding, or *via* bound H_2_O molecules.

In this study, we probe the effect of physiologically relevant intracellular pH values on the ATP binding affinity of the ε subunit from the monodirectional F_1_F_o_ ATP synthase from *C. thermarum*. Analysis of sequence and crystallographic data reveal two unique histidine residues may be responsible, with molecular dynamics (MD) simulations suggesting that the protonation state of these residues may influence ATP binding site stability.

## Results

### Unique histidine residues may be key players behind pH-guided affinity

**FIGURE 2:**
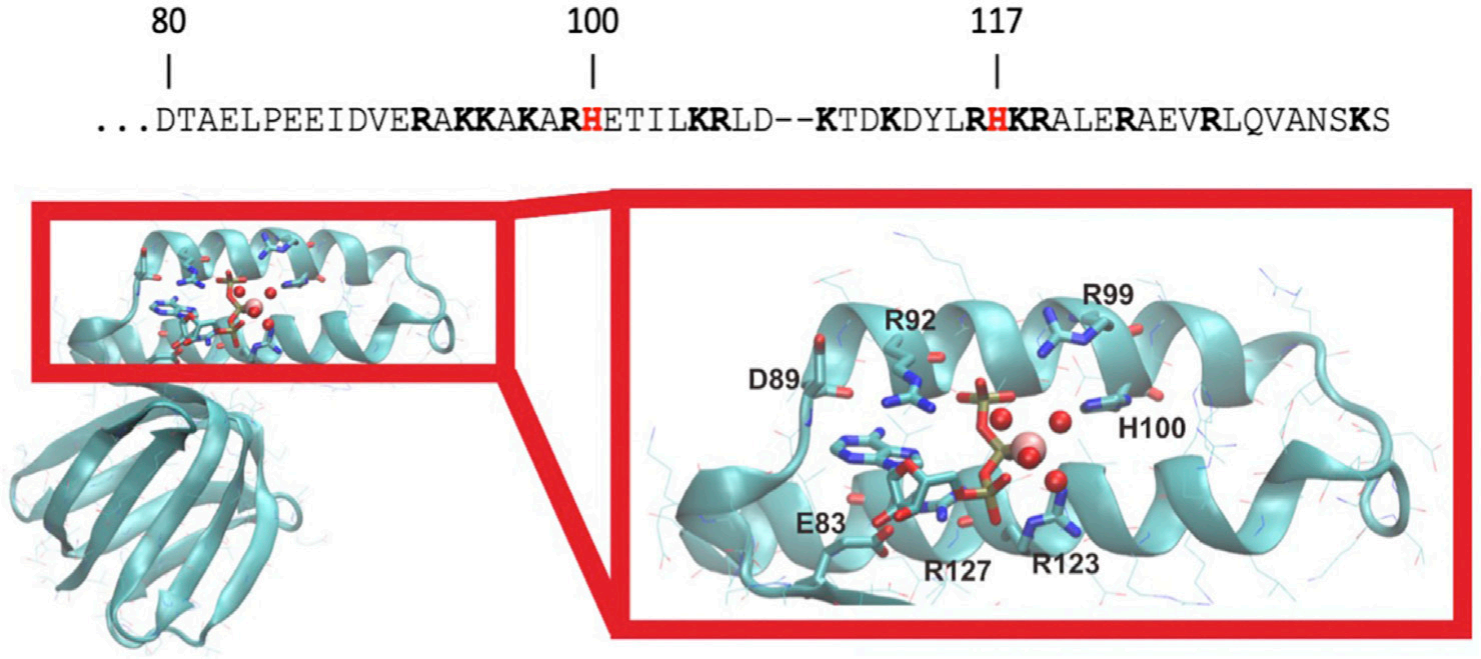
*C. thermarum* has unique histidine residues. The C-terminal helix amino acids of the ε subunit of *C. thermarum* (accession number are AFCE01000162) are shown with the unique histidine residues in red at positions 100 and 117 and other positive charged residues in bold type (top). Below this, a structural model of the ε subunit from *C. thermarum* in cyan (PDB ID: 5HKK) is displayed. The ligand-binding site structure is shown zoomed in on the right with the various features coordinating the ion highlighted. ATP is bound by εE83, εR92, εR99, εR123 and εR127. εH100 appears to stabilize Mg^2+^ (pink sphere) binding *via* one of the bound water molecules (red spheres). ATP is depicted as a stick model with its phosphates associating with Mg^2+^ and four water molecules.

**FIGURE 3:**
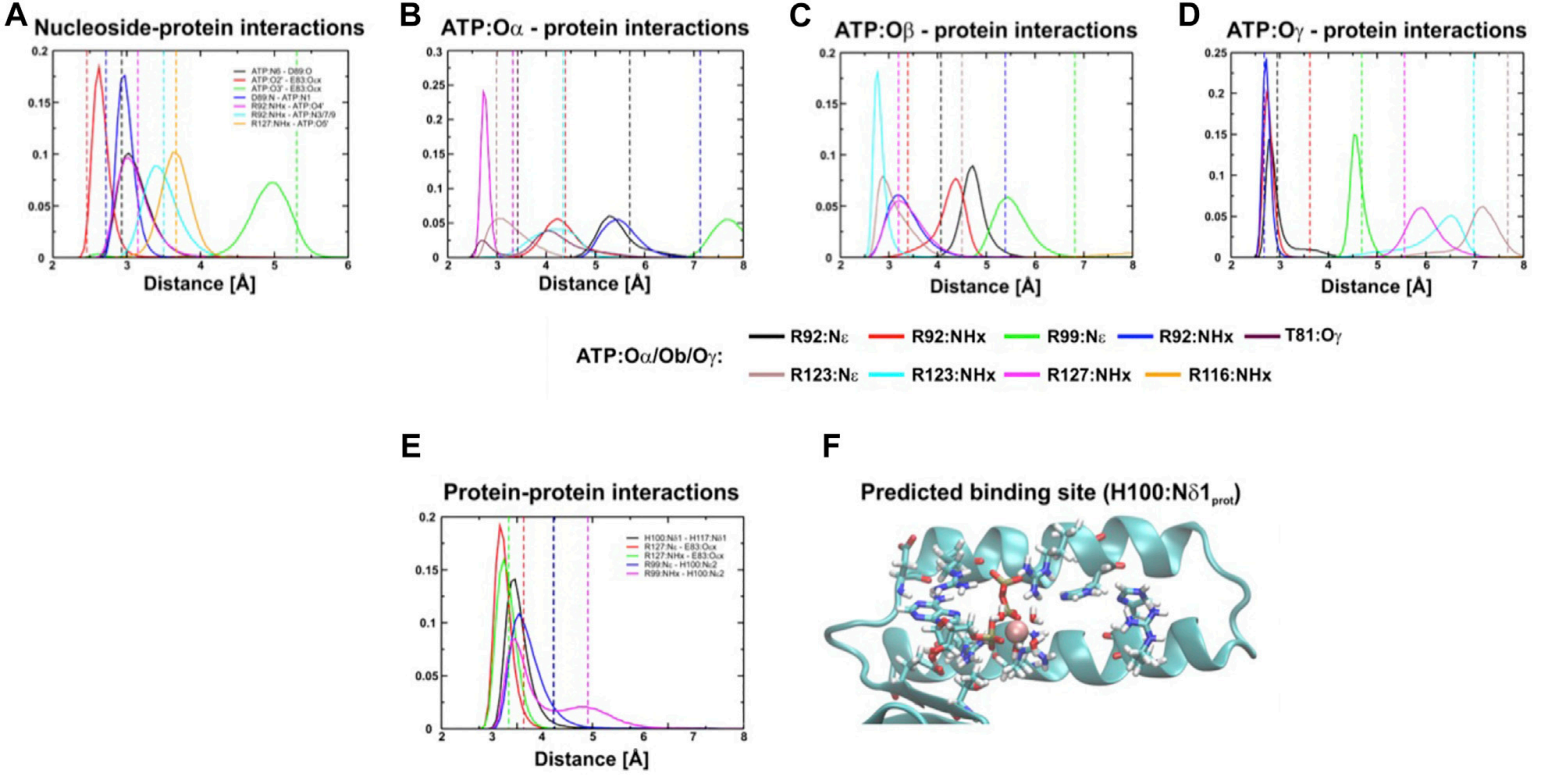
Distance distribution of interactions between the protein and ATP. **(A–E)**, Distance distribution of interactions within the binding site of the ε subunit (εH100:Nδ1 protonated) from *C. thermarum* during simulations. In **(F)** the predicted site is shown.

Initial examination of the *C. thermarum* ε subunit sequence in comparison with the neutrophilic *E. coli*, *Bacillus* PS3, *B. subtilis, Priesta megaterium* ε subunits and two ε subunits from the alkaliphilic *Alkalhalobacillus halodurans* and *Alkaliphilus psudofirmus*, revealed two unique histidine residues not present in any of the other species examined (Figure 2; Supplementary Figure S1). Fortunately, the crystal structure of the whole F_1_ domain from *C. thermarum* has been released recently (Ferguson et al., 2016), giving an opportunity to examine the relevance of these two histidine residues. Interestingly, the structure of the ε subunit with bound ATP and Mg^2+^ is quite similar to the ε subunit from thermophilic *Bacillus* PS3 (Yagi et al., 2007; Krah and Takada, 2016). ATP is coordinated by εE83 (ATP:O2ˊ), εD89:O (ATP:N6), εD89:N (ATP:N1), εR92 (cation-π stacking with the adenine base), εR99 (ATP:Oγ), εR123 (ATP:Oβ), εR127 (ATP:Oα/β) and one Mg^2+^ ion (Figures 2, 3). However, it should be noted that crystal packing effects may potentially have an influence on the structure (Krah et al., 2018).

### Exploring the potential role of the novel histidine residues using MD simulations

To help mitigate potential crystallographic effects and further explore the role of εH100 and εH117 we performed a series of triplicate MD simulations the ε subunit of *C. thermarum* as shown in the crystal structure (Ferguson et al., 2016). This revealed consistently stable binding of the protein to ATP over the timescale of hundred nanoseconds (Figure 3, Supplementary Figures S2–S5). The simulations indicated that the ATP binding site may be slightly shifted from the one observed in the crystal structure (Ferguson et al., 2016). Whether this conformation change reflects the presence of crystal contacts of the ε subunit with the α_3_β_3_ hexamer of the second crystallized ATP synthase is not clear (Krah et al., 2018). However, the computationally measured interactions are most stable if the proton of the εH100 imidazole ring in its neutral state is localized to Nδ1 (Figure 3). This means that the negative partial charge on Nε2 stabilizes the ATP binding site by supporting the εR99 interaction with the negatively charged γ-phosphate (Figure 3F). If εH100 is protonated only on Nε2, or double protonated on both Nδ1 and Nε2, we observed a decreased stability of the εR99:NHx–ATP:Oγ interaction.

In the crystal structure, hydrogen bonds are observed between εH100:Nδ1 and εH117:Nδ1. This indicates that the interaction with εH117 may also stabilize the position of H100; thus, the protonation state of εH117 may be of importance to induce a favourable interaction network and to prevent repulsion that may destabilize the εR99-εH100 and εR99-ATP:Oγ interactions. To test the influence of the protonation state on the stability, we thus also simulated different protonation states of εH100 and εH117. We then used the method of Espinosa et al. (1998) to assess the enthalpy associated with the hydrogen bond network, with the aim of examining if the conformation in which εH100:Nδ1 is protonated is indeed the most stable one (Table 1). Interactions were measured between ATP and protein, as well as between the CTH and remainder of the protein; the total energy shown is a sum of these two contributions. Our results demonstrate that the hydrogen bonding energy contributed by the network of interactions is more favourable for the εH100:Nδ1 protonation state than the εH100:Nε2 one (Table 1). The reduced stability in the latter case is likely caused by the loss of the hydrogen bond between εR99 and εH100 (Figure 3, Supplementary Figures S2–S4). Considering that the εR99 mutation in *Bacillus* PS3 lowers the ATP binding affinity (Kato et al., 2007), it may be expected that the pH changes the affinity due to a destabilized coordination network (Supplementary Figure S5).

**TABLE 1:** Energetic analysis of ATP binding to the ε subunit. Number of hydrogen bonds (H-bonds) and energetics (E_HB_) of ATP binding (enthalpic contribution) when the ε subunit from *C. thermarum* is simulated in different protonation states; the protonated imidazole nitrogen is indicated by Nδ1 (protonated at Nδ1), Nε2 (protonated at Nε2) or dp (double protonated). Interactions were measured between ATP and protein, as well as between the CTH and remainder of the protein; the total energy shown is a sum of these two contributions. Units of binding energy are reported in kcal/mol. Data is an average of 3 replica 100 ns simulations per system.


	**εH** **100**:**Nδ1/εH** **117**:**Nε2**	**εH100**:**Nε2/εH117**:**Nδ1**	**εH100**:**dp/εH117**:**Nε2**	**εH100**:**dp/εH117**:**dp**
No. H-bonds (protein-ATP)	11.7 ± 0.1	10.9 ± 0.2	11.2 ± 0.1	10.9 ± 0.1
No. H-bonds (protein-CTH)	6.4 ± 0.6	6.6 ± 0.2	6.6 ± 0.4	5.7 ± 0.4
	**εH100**:**Nδ1/εH117**:**Nε2**	**εH100**:**Nε2/εH117**:**Nδ1**	**εH100**:**dp/εH117**:**Nε2**	**εH100**:**dp/εH117**:**dp**
E_HB_ (H-bonds (protein-ATP))	−78.6 ± 0.7	−74.5 ± 0.8	−75.8 ± 1.6	−75.5 ± 0.7
E_HB_ (H-bonds (protein-CTH))	−35.0 ± 4.2	−37.1 ± 3.2	−37.3 ± 2.7	−30.8 ± 3.1
E_HB_ (total)	−113.6 ± 4.9	−111.6 ± 2.7	−113.1 ± 2.9	−106.2 ± 3.8

### ATP binding affinity is dependent on the pH

**FIGURE 4:**
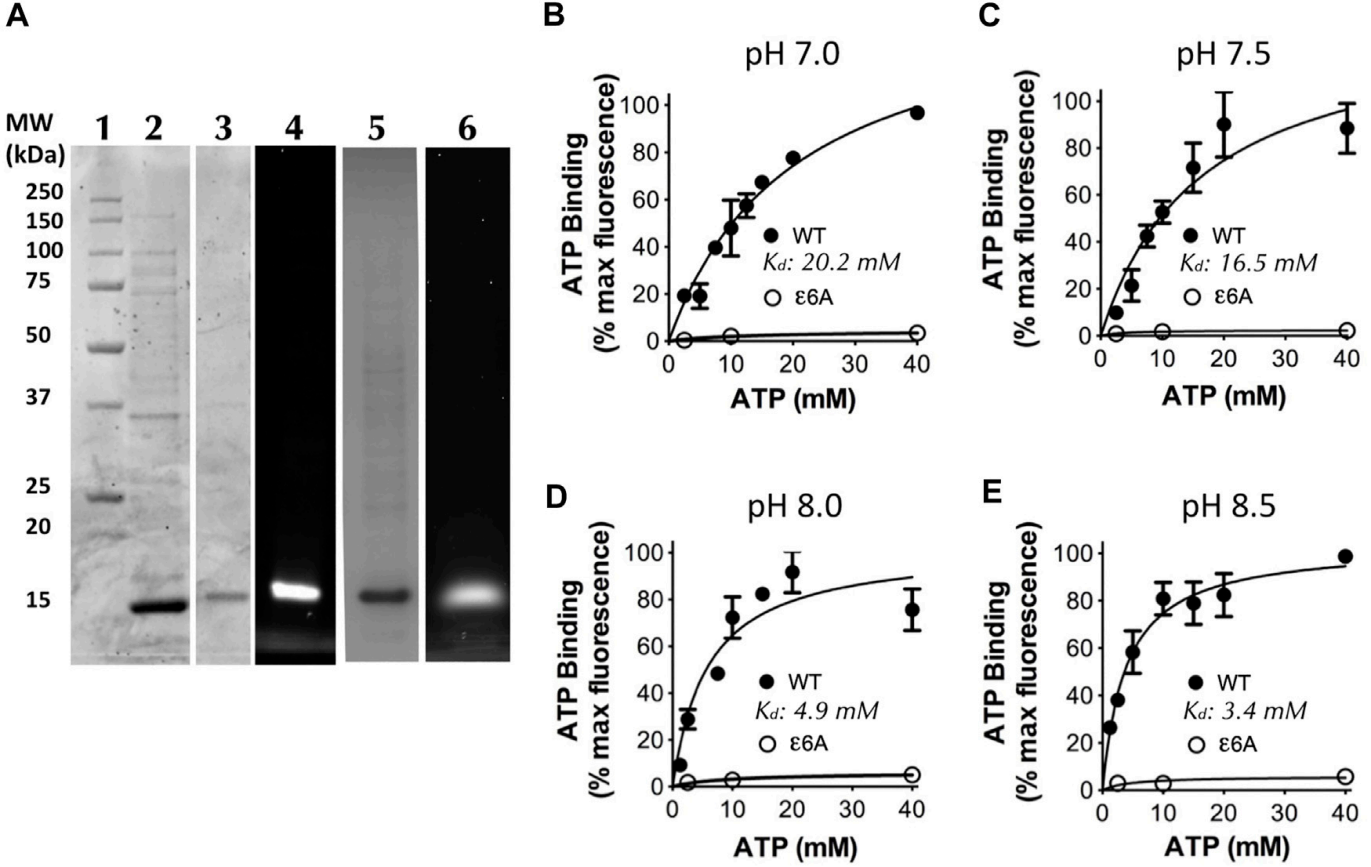
ATP binding by the *C. thermarum* ε subunit is regulated by pH. **(A)** SDS analysis of the ε subunit. Lane 1, Precision Plus protein standards (Biorad); Lane 2, as-purified *C. thermarum* ε subunit, Lanes 3 and 4, Cy3-labelled *C. thermarum* ɛ subunit visualized under either white light (Lane 3), or under a fluorescence emission filter at 590 nm (Lane 4). Lanes 5 and 6, Cy3-labelled *C. thermarum* ε6A mutant ε subunit visualized under either white light (Lane 5), or under a fluorescence emission filter at 590 nm (Lane 6). **(B–E)**: ATP binding curves using .4 μM Cy3-labelled *C. thermarum* ε subunit with hyperbolic 1-site fitting in the presence of equimolar Mg^2+^ at physiological relevant bulk-phase cell cytoplasmic pH values: **(B)** pH 7.0; **(C)** pH 7.5; **(D)** pH 8.0; or **(E)** pH 8.5. All results shown are the product of 3 experimental replicates.

To examine the ATP binding kinetics of the ε subunit from the *C. thermarum* F_1_F_o_ ATP synthase, we sub-cloned the wild-type (WT) ε subunit from the plasmid pATPHis5 (McMillan et al., 2007) and the CTH mutant ε6A (εR116A, εH117A, εK118A, εR119A, εR123A, and εR127A) from the plasmid pTrcF1e6A into the plasmid pET21. The ε6A mutant was selected due to its ability to confer ATP hydrolytic activity on the WT enzyme which was previously incapable of ATP hydrolysis (Keis et al., 2006). A cysteine residue was engineered into position 109 (εK109C), where it can be labelled to detect ATP binding, but is physically distant enough from the ATP/Mg^2+^ binding site as to not influence binding. Overexpression and purification of both WT and ε6A revealed a single band at approximately 14 kDa (WT: Figure 4A, Lane 2; ε6A: Lane 5) similar to the expected size for an isolated ε subunit from sequence data (de Jong et al., 2020). Labelling for ATP binding detection was achieved by specific attachment of cy3-maleimide modification of εC109 (WT: Figure 4A, Lanes 3 and 4; ε6A: Lane 6).

To examine the effect of changes in intracellular pH on ATP binding we measured the ATP binding affinity at a pH ranging from 7.0–8.5, spanning the possible local and bulk cytoplasmic pH range (de Jong et al., 2020) across the entire physiological growth spectrum of *C. thermarum* (McMillan et al., 2009). Interestingly, a strong effect of pH on the ATP binding affinity was revealed in two distinct clusters either side of pH 7.75. At pH 7.0 and 7.5 the *K*_*d*_ values were 20.2 and 16.5 mM respectively (Figures 4B, C), which is similar to that reported for *E. coli* (Yagi et al., 2007). However, at pH 8.0 and 8.5 the *K*_*d*_ values were 4.9 and 3.4 mM respectively (Figures 4D, E), similar to that of *B. subtilis* (Kato-Yamada, 2005; Krah et al., 2021). In contrast, the ε6A mutant did not bind any measurable ATP at any pH tested (Figures 4B–E). This confirms the ability of isolated *C. thermarum* ε subunit to bind ATP and shows that the affinity for ATP is strongly pH-dependent. The lack of ATP binding by the ε6A mutant implies that the ATP hydrolytic function that the ε6A mutations confer upon the *C. thermarum* enzyme is due to lack of ability to bind ATP. However, when reflecting upon this striking pH-dependent binding pattern, it is readily identified that the affinity is highest at the intracellular pH at which *C. thermarum* growth is fastest and it has the highest ATP synthesis ability; the optimal pH range is found between 9.0–9.5 (McMillan et al., 2009). It is also at this pH that the intracellular ATP concentration is highest, at 4 mM (Olsson et al., 2003), within the *K*_*d*_ range of the ε subunit. Yet when the extracellular pH drops to more acidic values of 8.5–7.5, the intracellular ATP drops with it to ∼2 mM, far below the *K*_*d*_ range of the ε subunit (Olsson et al., 2003). When reflecting upon the possible conformations of the ε subunit, it would be in the down-state precisely when it is synthesizing ATP and in the up-state when (if it could, but it does not) the organism intracellular pH decreases below optimal levels for cell function. This is precisely the opposite of what has been proposed for the ε subunit mode of action in *E. coli* and *Bacillus* PS3 using *in vitro* assays.

## Discussion

*C. thermarum* grows on various fermentable carbon sources as a facultative alkaliphile (pH 7.0–9.5), with an optimal growth rate at pH 9.5. However, on substrates such as malate and succinate, *C. thermarum* is an obligate alkaliphile, highly optimized for growth between pH 8.5 and 10.0 (McMillan et al., 2009). *C. thermarum* is unique, in that it is the only F_1_F_o_ ATP synthase to be described as mono-directional, only performing ATP synthesis unless chemically induced to perform the reverse reaction [ATP hydrolysis (McMillan et al., 2007)]. This is why ε subunit-mediated inhibition of ATP hydrolysis is such a fascinating aspect to study in this organism.

In this article, we present the first descriptive study that ascertains that ATP binding affinity of the ε subunit from the *C. thermarum* F_1_F_o_ ATP synthase is pH dependent; the binding affinity reduces remarkably at pH values less the 8.0. We propose this is due to its unique histidine residues helping to shape the architecture of the binding site. While these residues may be unique to *C. thermarum*, the tuning of ATP/Mg^2+^ affinity using second shell residues may indeed be the key behind unravelling the mystery of ε subunit-mediated regulation of ATP hydrolysis in the most import enzyme for energy generation in bacteria. Firstly, we consider how this might work from a chemical mechanism point of view, then from a biological impact aspect.

MD simulations indicate that this is likely caused by a protonation change of the histidine residue εH100 and εH117. Firstly, we consider the order of binding to the apo ε subunit.

**FIGURE 5:**
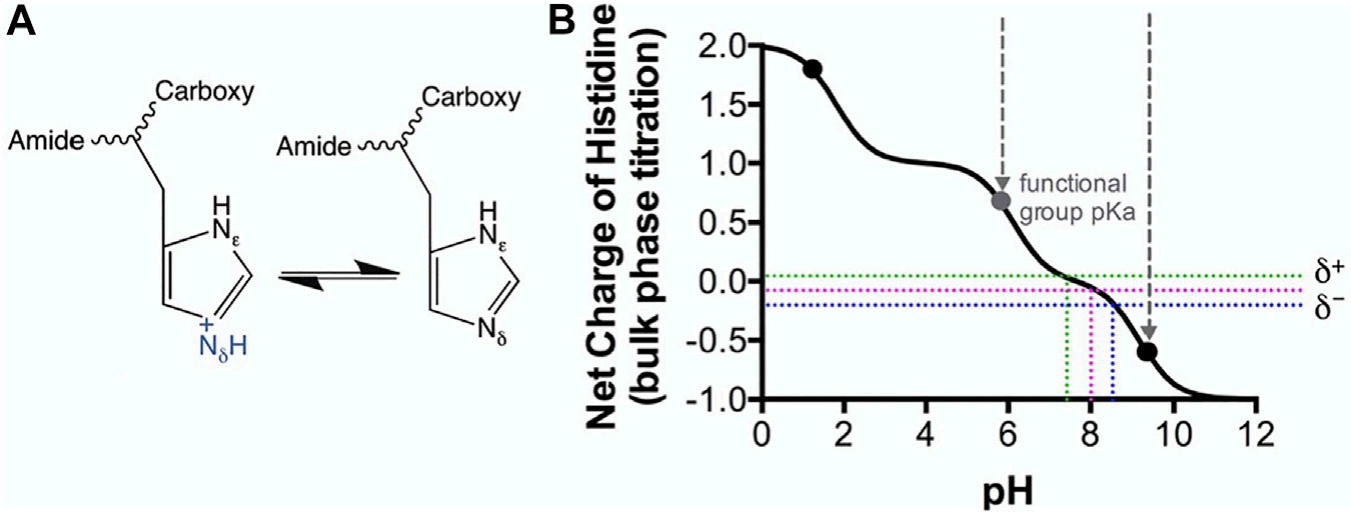
Modeled Histidine pKa shifts vs. pH. Histidine as a soluble amino acid was modeled vs pH using CurTiPot (Krah and Takada, 2016). **(A)** Lewis model of histidine chemistry showing gain and loss of a proton. **(B)** Model of histidine pK_a_ shift. Black dots indicate the pK_a_ values for histidine in solution. Perforated coloured lines indicate the pH values over which the histidine imidazole ring pK_a_ has a distribution between 1–0 net charge. pH values of 7.0 and 8.5 are indicated by the green and blue lines respectively. Perforated grey lines indicate the pH window of the functional group pK_a_.

Considering a solution phase pK_a_ of 6.0–6.5 for the imidazole group of a histidine residue (Tanokura et al., 1976) (Figures 5A, B), this structural feature may have crucial mechanistic influences derived by the environmental conditions. It is also noteworthy that this pK_a_ is only truly accurate for an amino acid in solution, in chemical isolation (an intrinsic “bulk-phase” pK_a_), and that the chemistry of the local structural environment, can shift; this is where H117 may also play a role. Since the histidine imidazole ring pK_a_ is a distribution between 1–0 net charge (between perforated green and blue lines, Figure 5B), it is reasonable to ascertain that some of the population of the ε subunit remains protonated up to just below pH 7.8, therefore reducing ATP binding affinity (Figure 5). It is indeed striking that the binding affinity changes 5.9-fold between a slight positive charge at pH 7.0 to a slight negative charge at pH 8.5.

To add further credence to this argument, εH100 is shown to an excellent resolution in the crystal structure of (Ferguson et al., 2016) for this type of analysis (2.6 Å) to coordinate *via* structurally bound H_2_O to a Mg^2+^ ion associating with ATP. This chemistry is not unlike that which is routinely utilized for purification in which a poly-histidine tag has affinity to Ni^2+^ or Co^2+^—as per manufacturers instruction. Strikingly, binding is well described to be most efficient at pH 8.0 and above where a slight negative charge is dominant (i.e., the histidine is neutral).

When considering physiology, the obligate aerobic alkaliphily of *C. thermarum* is completely coupled to aerobic cellular respiration. Oxygen consumption, succinate/malate transport, and ATP synthesis rates are all optimal at an external pH of 9.0–9.5 (McMillan et al., 2009). At the same time, constant internal pH is maintained between 7.8 and 8.5 (Olsson et al., 2003). Most pertinent to this study is that the cell ATP levels are highest when *C. thermarum* is growing at pH 9.0–9.5, and internal pH is 8.5 - at ∼4 mM. This is intriguingly close to the ATP/Mg^2+^ K_d_ values of the *C. thermarum* ε subunit we report here, suggesting the physiological relevance of the findings. Strikingly, when *C. thermarum* is grown at lower pH values than 9.0 the ATP concentration in the cell halves down to roughly ∼2mM, the intracellular pH drops to 7.8 (Olsson et al., 2003), and the affinity of the ε subunit for ATP decreases by ∼40% (see Figure 4D). This is very much in line with ATP synthesis experiments; due to the pK_a_-dictated proton capture by the *a* subunit, when extracelluar pH is below 8.5, ATP synthesis is drastically reduced (McMillan et al., 2007).

Clearly this is for a purpose–could the down-state of the ε subunit have a role in optimizing ATP synthesis in *C. thermarum*? And the up-state be a “molecular hand-break” ceasing all function? This seems in total contradiction to how the ε subunit in *E. coli* has been proposed to function. Yet we clearly demonstrate that when the internal pH drops below the physiological cytoplasmic pH of 7.8 it would appear ATP would not be bound to ε subunit, which would be in the extended up-state, inhibiting ATP hydrolysis/proton extrusion exactly when the cell would need it to extrude protons. Conversely, we observe higher affinity at the physiological cytoplasmic pH of 8.5, where the epsilon subunit would be in the ATP-bound down-state. This is exactly where one would expect that the up-state is needed to prevent wasteful ATP hydrolysis and enhance synthesis, if this was indeed its mechanism. The natural assumption at face value is that the ε subunit has no role in regulating this enzyme, as proposed by Ferguson *et al* (Ferguson et al., 2016) who suggested that ADP/Mg^2+^ is the sole regulatory element. Yet this assumption dismisses a prior study that clearly demonstrates that deletion of the C-terminal helix of the *C. thermarum* ε subunit results in “unlocking” of *in vitro* ATP hydrolytic activity in F_1_ preparations (Keis et al., 2006). We note that over the pH range examined in our study, the ATP hydrolysis rates in isolated F_1_F_o_ ATP synthase are actually higher at pH 7.5 than pH 8.0 (McMillan et al., 2007). However; a) the enzyme does not natively hydrolyse and b) LDAO is confounding such a comparison. The key to this apparent conundrum may be in the role of magnesium in this enzyme and is a subject for further studies.

## Materials and methods

### Molecular dynamics simulations and analysis

MD simulations were carried out using the ε subunit (chain H, PDB ID: 5HKK) from the *C. thermarum* TA2.A1 F_1_F_o_ structure and the ligands bound to the protein (ATP, Mg^2+^ and four water molecules) (Ferguson et al., 2016). To predict the protonation states of titratable residues, we applied the H++ webserver (Anandakrishnan et al., 2012), except for εH100 and εH117. Because εH100 is located near the ATP binding site and εH117 is interacting with this residue, we set up four different protonation and thus coordination states (εH100Nδ1/εH117Nε2, εH100Nε2/εH117Nδ1 and εH100dp/εH117Nε2, εH100dp/εH117dp). The protein was solvated and three additional Mg^2+^ ions were added, as described previously (Krah and Takada, 2016). Counter ions (Cl^−^) were also added to neutralize the simulation systems. To obtain sufficient sampling we carried out three independent simulations of each εH100/εH117 protonation state. Each system was equilibrated for 4 ns, gradually releasing the strength of position restraints applied to the protein every 1 ns, followed by 100 ns of unrestrained production runs.

To carry out the simulations, we used the GROMACS (v. 5.1.2) program suite (Abraham et al., 2015), applying the AMBER-ILDN force field (Cornell et al., 1995; Wang et al., 2000; Meagher et al., 2003; Hornak et al., 2006; Lindorff-Larsen et al., 2010) as implemented (Sorin and Pande, 2005) in GROMACS. We used Mg^2+^ ion parameters (Ȧqvist, 1990) as described previously. Pressure and temperature were kept constant at 1 bar and 300 K, utilizing the Parinello-Rahman barostat (Parrinello and Rahman, 1981) and v-rescale thermostat (Bussi et al., 2007), respectively. We applied an integration time step of 2 fs. Electrostatic interactions were calculated using the Particle Mesh Ewald (PME) method with a real-space cut-off of 12 Å. Calculations of the van der Waals interactions was carried out using the same cut-off. Periodic boundary conditions were applied in all directions.

We estimated the ligand binding enthalpy for the contracted down state using the method introduced by Espinosa et al. (1998). The analysis includes protein-nucleotide and protein-protein interactions, reflecting the hydrogen bonds between the CTD (residues 112–134), which undergoes the conformational change, and the rest of the protein. The hydrogen–acceptor distance was set to a maximum of 2.7 Å, and the associated angle to 30°. Molecular structures were visualized using VMD (Humphrey et al., 1996) and the sequence alignment in Supplementary Figure S1 was done with Jalview (Waterhouse et al., 2009).

### Cloning of the *C. thermarum* TA2.A1 wildtype ε subunit and ε6A mutant

Wildtype ε subunit and ε6A mutant ε subunit (εR116A, εH117A, εK118A, εR119A, εR123A, and εR127A) from the *C. thermarum* TA2.A1 F_1_F_o_ ATP synthase (henceforth referred to as ‘WTε and ε6A subunits’) were subcloned from pATPHis5 (McMillan et al., 2007), pTrcF1e6A (Keis et al., 2006) by polymerase chain reactions (PCR). During PCR *Hin*dIII and *Nde*I sites were introduced with primers TA2_Eps_Fw and TA2_Eps_Rv, (see Table S1). The PCR reaction mixture contained Q5^®^ Hot Start High-Fidelity 2X Master Mix (New England Biolabs), 10 p.m. of each primer and 10 ng DNA. Amplification was performed in a Biometra TAdvanced PCR machine (Analytik Jena), using the following program: 30 s 98 °C, 30x (5 s 98 °C, 30 s. 72 °C), 30 s. 72 °C. The 1.4 kb PCR products were cleaned using the Monarch^®^ PCR and DNA Cleanup Kit (New England Biolabs) and digested with *Hin*dIII, *Nde*I and *Dpn*I restriction enzymes (New England Biolabs). We then digested 1 µg plasmid pET21-BsF1epsilonQ107CE102A (Krah et al., 2021) DNA with 1U of both *Nde*I and *Hin*dIII in CutSmart^®^ Buffer (New England Biolabs). Digested inserts and plasmid backbone were separated from unwanted DNA fragments using a 1% agarose gel, and the desired fragments purified from the gel using a gel extraction kit (Qiagen). The plasmid backbone (∼200 ng) and either insert (∼100 ng WT ε or ε6A mutant) were then ligated together with T4 DNA ligase and T4 DNA ligase buffer (New England Biolabs) for 1 h at room temperature and then inactivated for 10 min at 65 °C. 1 µL of ligation mixture was transformed to chemical competent *E. coli* DH5α strains and plated onto LB-agar plates containing .1 g/L ampicillin. Colonies containing the insert were grown in 5 mL εLB medium containing .1 g/L ampicillin and plasmid was extracted from these cultures. The plasmids were sent to Baseclear (Leiden) for sequencing using the T7 and T7-R primers for verification. The new plasmids containing either WTε and ε6A subunits are named pET21-CthF1ε and pET21-CthF1ε6A, respectively.

### Side directed mutagenesis

For introduction of the cysteine point mutation to enable cy3 labelling of the WTε and ε6A subunits, the Quikchange procedure was followed using the TA2_K109C_Fw and TA2_K109C_Rv primers (see Table S1) targeting the sequence of the WTε and ε6A subunits in pET21-CthF1ε and pET21-CthF1ε6A resepctively. The PCR mixture of contained 10 pmol of each primer, 10 ng μL either pET21-CthF1ε or pET21-CthF1ε6A plasmid DNA and Q5^®^ Hot Start High-Fidelity 2X Master Mix. The amplification was performed using the following program: 30 s 98 °C, 30x (20 s 98 °C, 50 s 60 °C and 420 s 72 °C), 30 s 72 °C. 5 µL of PCR mixture was removed and 0.5U of *Dpn*I and 4 µL of CutSmart^®^ Buffer was added. After 2 h of incubation at 37°C the sample was deactivated for 15 min at 65°C. 1 µL of mixture was transformed to chemical competent *E. coli* DH5α strains and plated onto LB-agar plates containing .1 g/L ampicillin. A colony PCR was performed (using the cloning primers from before) to verify the presence of the insert. The PCR product was loaded on a 1% agarose gel containing .01% SYBR safe. Colonies containing the correct plasmid were grown in 5 mL LB medium containing 0.1 g/L ampicillin and plasmid was extracted from these cultures. The plasmid was sent to Baseclear (Leiden) for point mutation verification using the T7 and T7-R primers for verification. The new plasmids containing either the wildtype ε or ε6A mutant subunits of *C. thermarum* TA2.A1 F_1_F_o_ ATP synthase with the K109C point mutation are named pET21-CthF1ε,K109C or pET21-CthF1ε6A,K109C plasmid DNA. From here on referral to *C. thermarum* TA2.A1 WTε and ε6A subunits will be εK109C mutants.

### Overexpression and purification

Overexpression of *C. thermarum* TA2.A1 WTε and ε6A subunits was conducted in a method based on Krah et al., 2021 (Krah et al., 2021). *Escherichia coli* BL21 (DE3) cells containing either pET21-CthF1ε,K109C or pET21-CthF1ε6A,K109C were grown on 2x YT medium with .1 g/L ampicillin and 2 g/L glucose at 37 °C and 180 rpm. When an OD_600_ value of >.5 was reached overexpression was induced by the addition of 0.1 mM filter-sterilized 0.2 mM isopropyl β-D-1-thiogalactopyranoside (IPTG, Thermo Fisher Scientific). The cultures were then cultivated for a further 3 h at 37°C and 180 rpm after which the cells were harvested by centrifugation at 9,000 × *g* for 10 min and cell pellets resuspended in 50 mM Tris/HCl (pH 7.5), 1 mM MgCl_2_. A cocktail of fresh .1 mM PMSF (phenylmethylsulfonyl fluoride, Sigma Aldrich), 0.1 mg/mL deoxyribonuclease I (Sigma Aldrich), and 1 mM fresh DTT (Sigma Aldrich) was added immediately before lysis by two passages through a cell disruptor at 1.8 kbar (Constant Systems). The lysate was centrifuged at 9,000 × *g* for 10 min. The ε subunit was in inclusion bodies, so was resuspended in 20 mL 50 mM Tris/HCl (pH 7.5), 2 mM EDTA and 1 mM fresh DTT (buffer 1). The resulting pellet was resuspended and centrifuged at 9,000 × *g* for 10 min yielding soluble ε subunit in the supernatant. This wash step was repeated 5 times before the supernatant was concentrated with Amicon Ultra 3K filters (Merck) to a final volume of 200 µL. The ε subunit was then further purified with a Superdex 200 Increase 10/300 GL (GE Healthcare Life Sciences) size exclusion chromatography column with an NGC System (Biorad). The column was equilibrated with 10 mM Tris/HCl (pH 7.5), 2 mM EDTA and 140 mM NaCl (buffer 2) before sample was injected in the column. Thereafter, the column was eluted with 1.25 CV of buffer 2 supplemented with 1 mM DTT. Eluent fractions containing ε subunit were concentrated, frozen with liquid nitrogen and stored at −80°C.

### Protein determination and SDS-PAGE

Protein concentration was determined using the Bicinchoninic acid assay (Sigma) according to the manufacturer’s instructions using bovine serum albumin as a standard. Protein samples were analyzed with SDS-PAGE using 4%–12% Criterion™ XT Bis-Tris Protein Gel then stained with SimplyBlue™ SafeStain (Invitrogen).

### Fluorescent labeling of ε subunits

The *C. thermarum* ε subunits were labeled as decribed previously in Krah et al., 2021 (Krah et al., 2021). Both ε subunit samples were desalted with a PD Minitrap™ G-25 (GE Healthcare) equilibrated with 50 mM HEPES-NaOH (pH 6.5) and 100 mM NaCl. TCEP was added in a 5:1 protein molar ratio to the ε subunit samples and they were incubated for 2 h at 25°C. Cy3 maleimide in dimethylsulfoxide (DMSO) was then added in a 5:1 dye to protein molar ratio and the mixture incubated for a further 2 h at 25°C. temperature. Excess dye was removed with the same centrifuge column equilibrated with 50 mM HEPES-KOH (pH 7.5), 100 mM KCl, 10 mM MgCl_2_. Labeling was subsequently analyzed by SDS-PAGE and Cy3 maleimide labeling detected by imaging the unstained gel with an Amersham Typhoon Imaging System (GE Healthcare). Gels were subsequently stained with SimplyBlue™ SafeStain (Invitrogen).

### ATP binding assays

ATP binding was detected *via* fluorescence using a Synergy 2 Microplate Reader (Biotek). Cy3 maleimide fluorescence was measured at 22°C during ATP binding to either *C. thermarum* ε subunit or the ε6A variant, in a system with an excitation filter of 530 nm (25 nm bandwidth) and an emission filter at 590 nm (35 nm bandwidth). Each reaction mixture contained 800 nM labeled ε subunit in 50 mM MOPS-Tris buffer (either pH 7.0, 7.5, 8.0, 8.5), 100 mM KCl and equimolar MgCl_2_:ATP in a final volume of 195 µL. Fluorescence was measured for 30–60 s at 1.66 Hz followed by an injection of concentrated 5 µL ATP in the same buffer. This resulted in a final labeled ε subunit concentration of 400 nM. Fluorescence was measured over the course of the experimental observation window for 6 min at 1.66 Hz.

## Data Availability

The raw data supporting the conclusion of this article will be made available by the authors, without undue reservation.
