# Supplementary material for: ATP binding by an F1Fo ATP synthase ε subunit is pH dependent, suggesting a diversity of ε subunit functional regulation in bacteria
Source: Front Mol Biosci. 2023 Feb 27;10:1059673. doi: 10.3389/fmolb.2023.1059673 (PMC10010621; doi:10.3389/fmolb.2023.1059673)
Supplement: Supplementary file 1 [file Table1.DOCX]

**SUPPLEMENTARY MATERIAL FOR**

**ATP binding by an F_1_F_o_ ATP synthase ε subunit is pH dependent, suggesting a diversity of ε subunit functional regulation in bacteria.**

Alexander Krah^§1,2*^, Timothy Vogelaar^3^, Sam I. de Jong^3^, Jolyon K. Claridge^4^, Peter J. Bond^2,5^ and Duncan G.G. McMillan^§3,4*^

^§^These authors contributed equally to this manuscript

^1^Korea Institute for Advanced Study, School of Computational Sciences, 85 Hoegiro, Dongdaemun-gu, Seoul, 02455, Republic of Korea

^2^Bioinformatics Institute, Agency for Science, Technology and Research (A*STAR), 30 Biopolis Str., #07-01 Matrix, Singapore 138671

^3^Delft University of Technology, Department of Biotechnology, Van der Maasweg 9, Delft 2629HZ, The Netherlands

^4^Massey University, School of Fundamental Sciences, Tennent Drive, Palmerston North 4472, New Zealand

^5^National University of Singapore, Department of Biological Sciences, 14 Science Drive 4, Singapore 117543

*Contact: [D.G.G.McMillan@tudelft.nl](mailto:D.G.G.McMillan@tudelft.nl) (DGGM) or [kraha@](mailto:alexkrah.akad@)bii.a-star.edu.sg (AK)


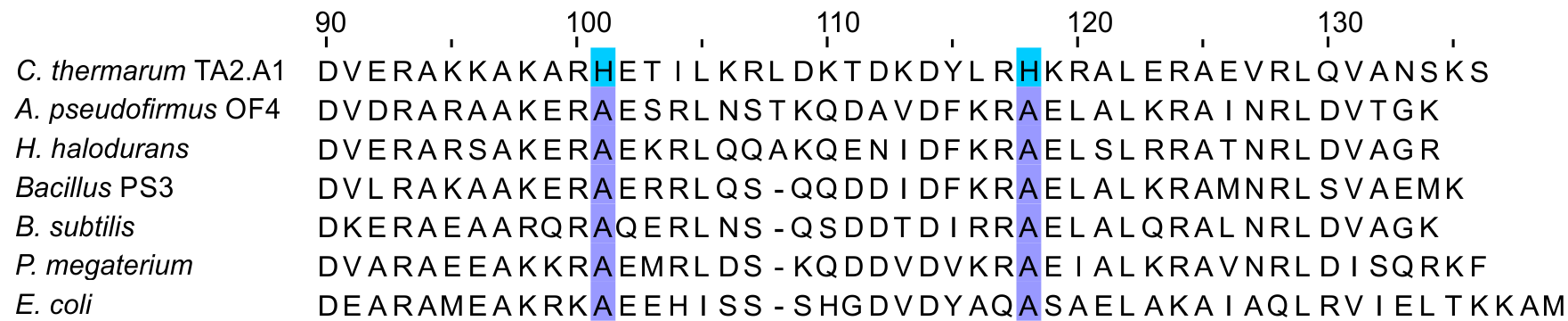


Figure S1: Alignment of the C-terminal helicies of various bacterial epsilon subunits from F_1_F_O_-ATP synthases. Alignment of the ε subunits of alkaliphilic *Caldalkalibacillus thermarum* TA2.A1 (PDB-ID: 5HKK), *Alkaliphilus pseudofirmus* OF4 (Uniport-ID: P22480), *Alkalhalobacillus halodurans* (Uniport-ID: Q9K6H6), *Bacillus* sp. PS3 (PDB-ID: 2E5Y), *Bacillus subtilis* (Uniprot-ID: P37812)*, Priestia megaterium* (Uniprot-ID: P12699) and neutrophilic *Escherichia coli* (PDB-ID: 1AQT). The ε subunit from alkaliphilic *Caldalkalibacillus thermarum* TA2.A1 revealed two unique histidine residues (in cyan) not present in any of the other species examined.


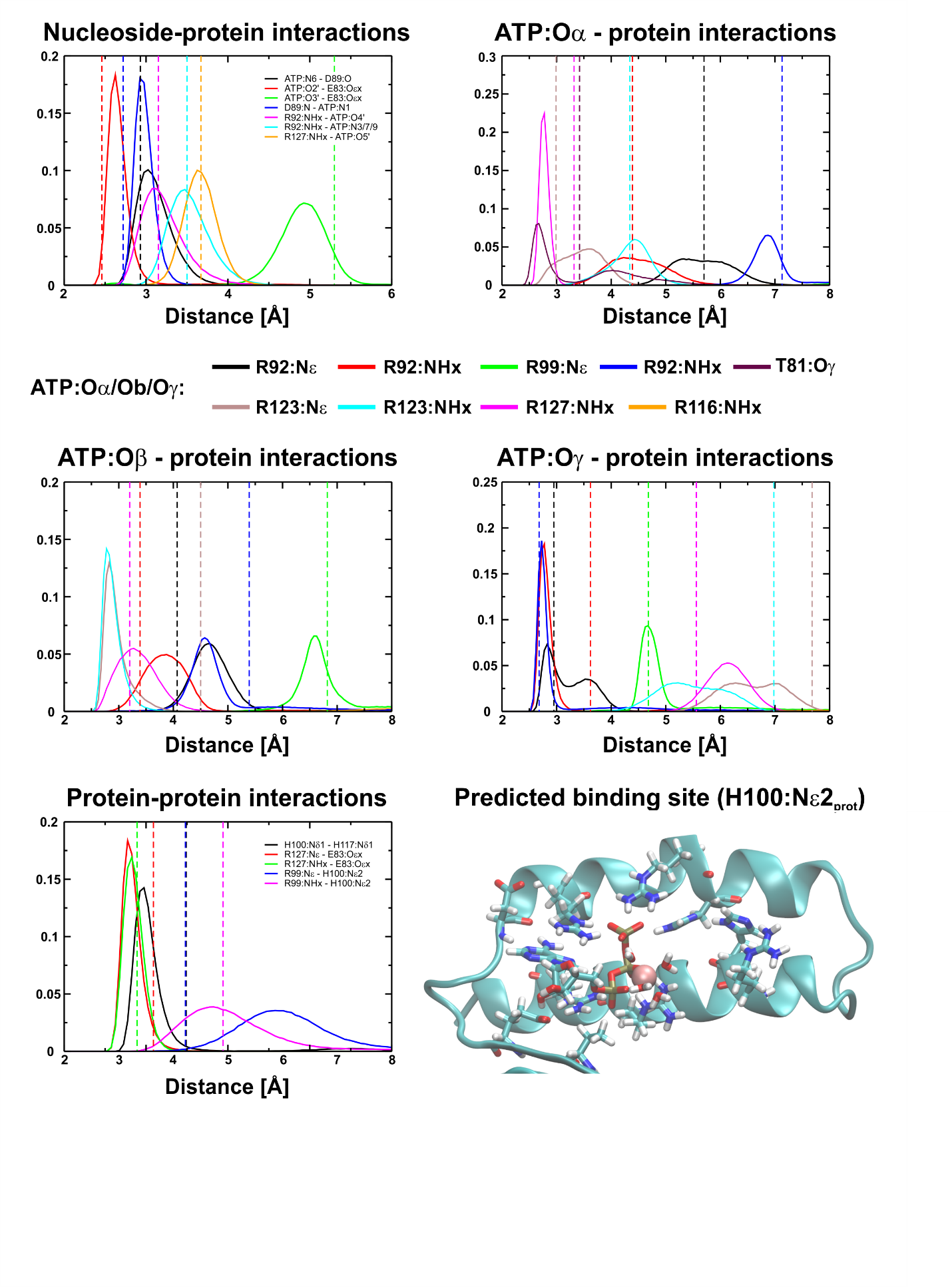


Figure S2: Distance distribution of interactions within the binding site of the ε subunit (H100:Nε2 protonated) from *Caldalkalibacillus thermarum* TA2.A1 during simulations. The predicted site is shown in the bottom right Figure.


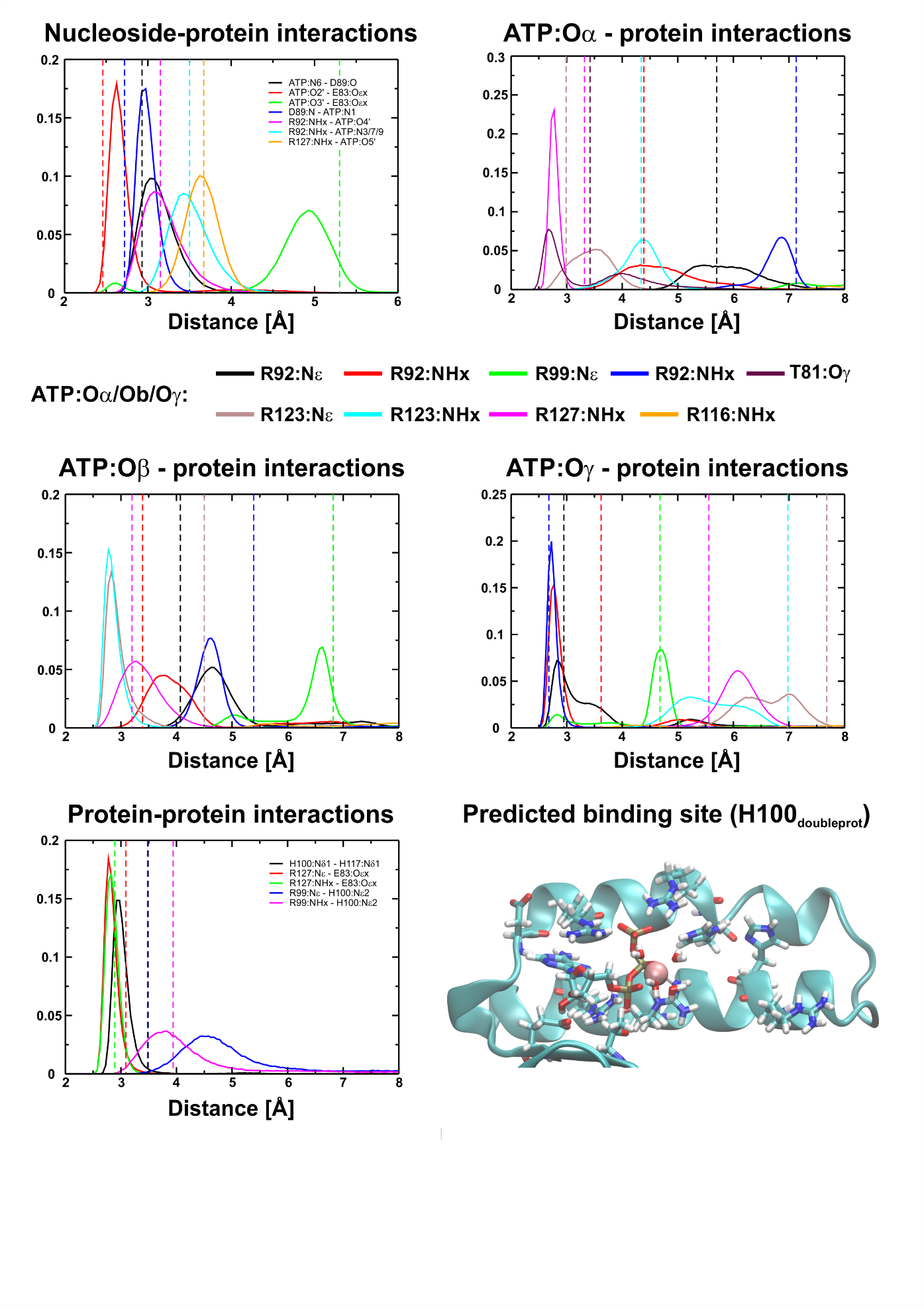


Figure S3: Distance distribution of interactions within the binding site of the ε subunit (H100 double protonated) from *Caldalkalibacillus thermarum* TA2.A1 during simulations. The predicted site is shown in the bottom right Figure.


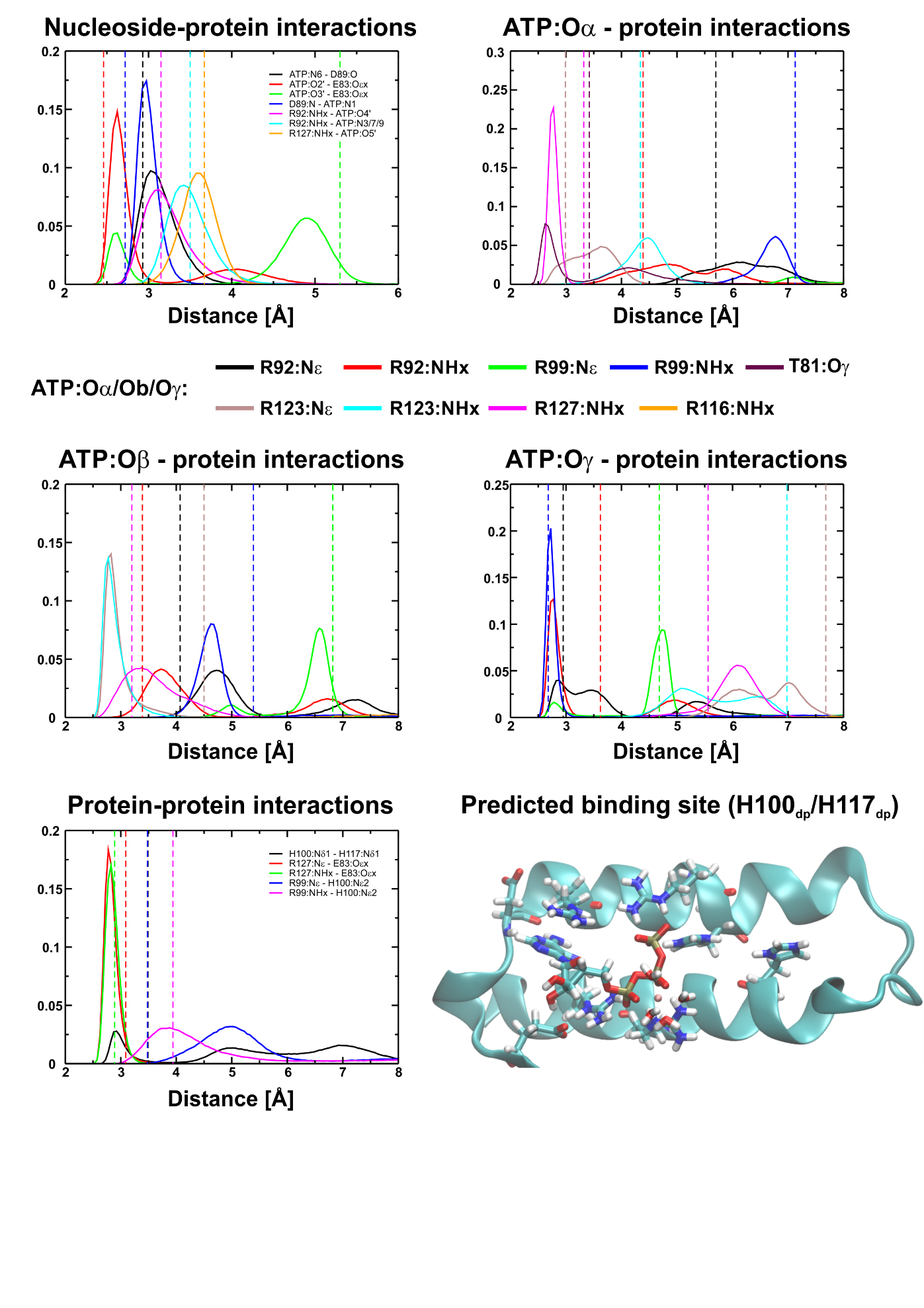


Figure S4: Distance distribution of interactions within the binding site of the ε subunit (H100 and H117 double protonated) from *Caldalkalibacillus thermarum* TA2.A1 during simulations. The predicted site is shown in the bottom right Figure.


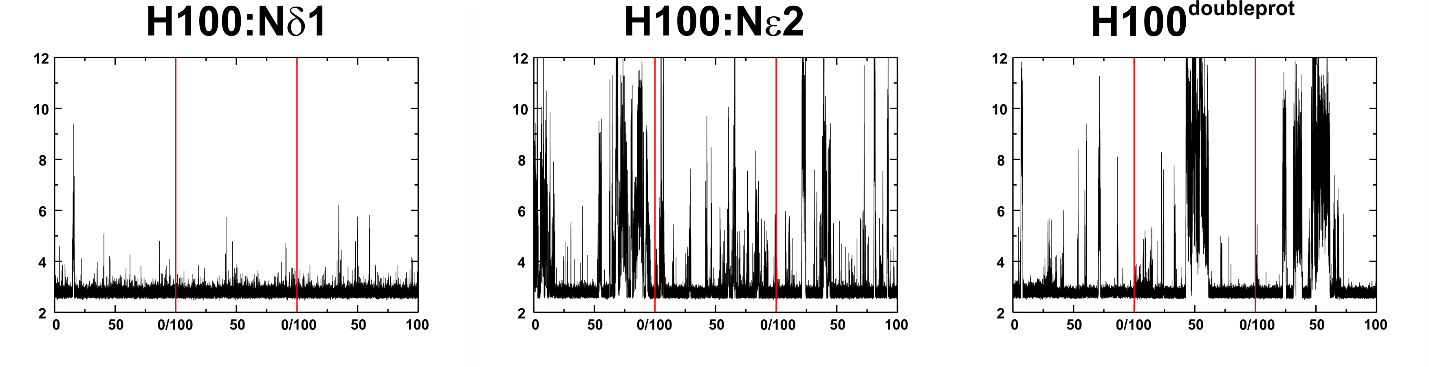
Figure S5: Distance of R99:NHx to ATP:Oγ. A decreased stability of this interaction if H100 is protnated at Nε2 or if it is double protonated. The Figure show the results of three independent runs; the single runs are divided by a red line.


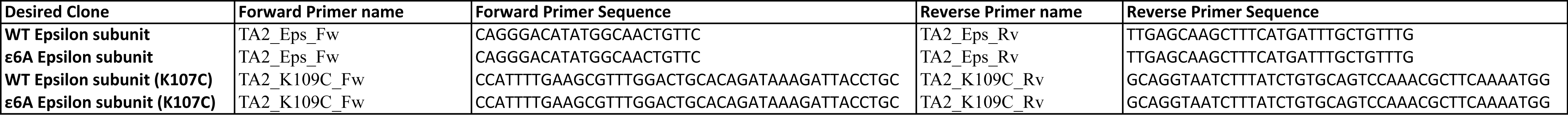


Table S1: Primers used in this study (shown 5’ to 3’)
